# Validation of a Lumped Parameter Model of the Battery Thermal Management System of a Hybrid Train by Means of Ultrasonic Clamp-On Flow Sensor Measurements and Hydronic Optimization

**DOI:** 10.3390/s23010390

**Published:** 2022-12-30

**Authors:** Raffaele De Rosa, Luca Romagnuolo, Emma Frosina, Luigi Belli, Adolfo Senatore

**Affiliations:** 1 Department of Industrial Engineering, University of Naples “Federico II”, Via Claudio, 21, 80125 Napoli, Italy; 2 Department of Engineering, University of Sannio, Via Roma 21, 82100 Benevento, Italy; 3 Hitachi Rail STS S.p.A., Via Argine 425, 80147 Napoli, Italy

**Keywords:** battery thermal management system (BTMS), hybrid train propulsion, ultrasonic flow sensor, clamp-on sensor, hydronic balancing methods

## Abstract

Electrification of the field of transport is one of the key elements needed to reach the targets of greenhouse gas emissions reduction and carbon neutrality planned by the European Green Deal. In the railway sector, the hybrid powertrain solution (diesel–electric) is emerging, especially for non-electrified lines. Electric components, especially battery power systems, need an efficient thermal management system that guarantees the batteries will work within specific temperature ranges and a thermal uniformity between the modules. Therefore, a hydronic balancing needs to be realized between the parallel branches that supply the battery modules, which is often realized by introducing pressure losses in the system. In this paper, a thermal management system for battery modules (BTMS) of a hybrid train has been studied experimentally, to analyze the flow rates in each branch and the pressure losses. Since many branches of this system are built inside the battery box of the hybrid train, flow rate measurements have been conducted by means of an ultrasonic clamp-on flow sensor because of its minimal invasiveness and its ability to be quickly installed without modifying the system layout. Experimental data of flow rate and pressure drop have then been used to validate a lumped parameter model of the system, realized in the Simcenter AMESim^®^ environment. This tool has then been used to find the hydronic balancing condition among all the battery modules; two solutions have been proposed, and a comparison in terms of overall power saved due to the reduction in pressure losses has been performed.

## 1. Introduction

The transport sector is one of the main causes of CO_2_ emissions [1], and trains are responsible for 4.6% of the greenhouse gas emissions from transportation because, in several countries, there are still non-electrified lines with trains powered by diesel engines [2]. Electrification costs time and money; battery power, on the other hand, is the cleanest zero–emission solution to replace diesel trains and start achieving climate change targets, which will instantly improve air quality in cities and non-electrified stations. This technology will allow for travel beyond electrified routes, ensuring seamless journeys [3]. Moreover, battery-powered trains can also use regenerative braking, making them much more environmentally friendly than diesel railcars [4].

However, a battery power system is a temperature-sensitive technology, where performance is influenced by the temperature in terms of efficiency, lifetime, and safety [5,6]. For lithium-ion batteries, the optimum operating temperature is between 20 and 40 °C, and the temperature difference between battery cells of the same pack should be less than 5 °C. Furthermore, the improper distribution of temperature can cause significant performance degradation and may also lead to overheating and thermal runaway [7,8,9]. For these reasons, the battery thermal management system (BTMS) is one of the most crucial elements of an electric train. Air cooling, liquid cooling, and heat pipe-based cooling are the most common principles on which the BTMS is built [10,11,12,13,14,15], but there are also new technologies such as phase change material-based cooling [16] and thermoelectric element-based cooling [17,18,19,20].

Unlike other sectors, such as automotive and aerospace, where it is by now consolidated, battery-based electric powertrain in the railway field is still in the embryonic stage. Only in recent years, due to the Sustainable and Smart Mobility strategy [21] that is encouraged by the EU and other countries, researchers and big players are developing a program that involves removing diesel engines and replacing them with a battery-based electric powertrain to operate on non-electrified lines [3,22,23]. Those systems must be correctly controlled in terms of temperature, especially the battery pack. Studies on BTMS have also been widely developed in fields such as the automotive and aerospace. Xiong et al. [24] developed an AMESim model of a liquid cooling system for a power battery of a plug-in hybrid electric vehicle in order to analyze the thermal behavior. Sun et al. [16] conducted a numerical analysis into the inhibiting effects of a novel hybrid BTMS, combining active and passive cooling on thermal runaway propagation caused by single cell. Kellerman et al. [25] developed a numeric model of BTMS for a hybrid electric aircraft under the assumption that the ambient temperature may be higher than the allowed battery operating temperature.

In the railway field, a different approach is needed, especially with regard to liquid cooling systems. Batteries in the railway applications must satisfy an energy accumulation in the order of MWh [26] while, in the automotive sector, it is in the order of tens of kWh [27,28]. For this reason, in railway applications, the overall dimension of the battery packs is much larger and the refrigerant flow rate requirement is also higher. Therefore, given the large flow rates involved, hydraulic optimization can lead to considerable energy savings.

Previous research on BTMS for battery powered train mainly focused on thermal aspects. Iwase et al. [4] conducted a thermal simulation to confirm the feasibility of natural air cooling for train battery storage systems. Kang et al. [29] proposed a thermal prediction model of a 1S18P battery pack classified into joules heating with equivalent resistance, reversible heat, and heat dissipation. Teng and Yeow [30] analyzed the thermal performance of two battery module cooling methods with the indirect liquid cooling system with three types of tubular cooling plates between cells with interior fluid; they concluded that the structural layout of a multiple parallel-channels cold plate resulted in a lower coolant pressure and temperature gradient.

The aim of this research is the study of a liquid cooling system for train batteries, which goes from the inlet manifold to the outlet manifold of the hydraulic system and to optimize it from a hydronic point of view in order to find the condition that entails the balancing of the flow rate in all the branches, to minimize the pressure drops and to guarantee to all modules have the same operating conditions. To achieve this goal, a BTMS is studied experimentally and numerically. From the results of the experimental campaign, the Lumped Parameter Model (LPM) is validated. LPMs simplify spatially distributed systems into discrete entities, in which radial or axial gradients are not considered and interactions with the surroundings occur through ports on the boundary. The key advantages of this approach are computational efficiency and simplification of the mathematical formulation, even for more complex systems. However, in some cases the 0D-1D approach alone is not sufficient, and, for this reason, 3D modeling is required for some system components. The validation takes place in two phases. The first involves the validation of the hydraulic system without batteries. The second provides the hydraulic characterization of the battery module on the bench. Thanks to the validated model, numerical tests are then carried out that are aimed at finding the aforementioned conditions. These are described in detail in Section 4.2.

This paper follows the steps of the LPM first introduced in [31], in which the priority was the cooling of the power converters. In this case, our study focuses on the hydronic balancing of the liquid cooling system for battery modules.

## 2. Materials and Methods

### 2.1. System Architecture and Description

**Figure 1:**
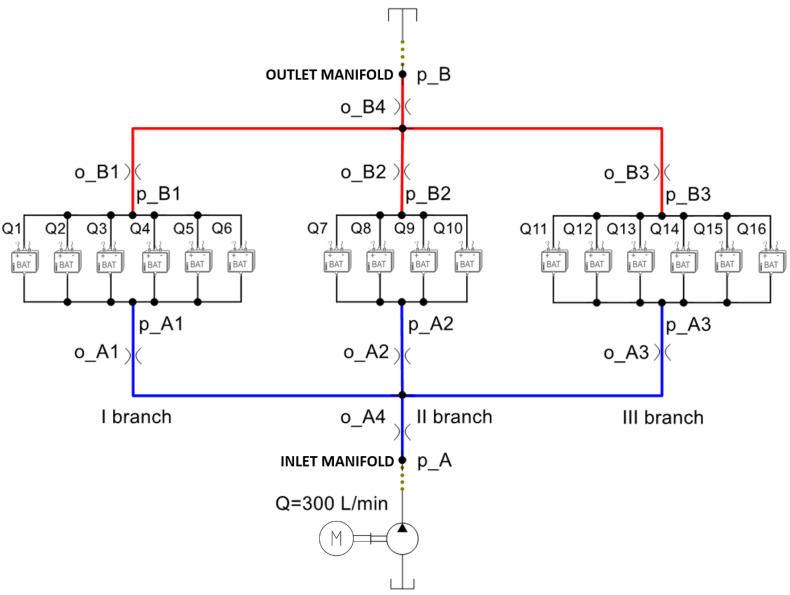
Battery liquid cooling system.

The cooling system represented in Figure 1 shows a battery box made of sixteen battery modules arranged in three parallel branches, in order to obtain the best performance. Each module must be cooled by the same coolant flow rate and supplied at a constant temperature; therefore, the fluid is first cooled in a chiller and then supplied to the modules at 25 °C.

**Figure 2:**
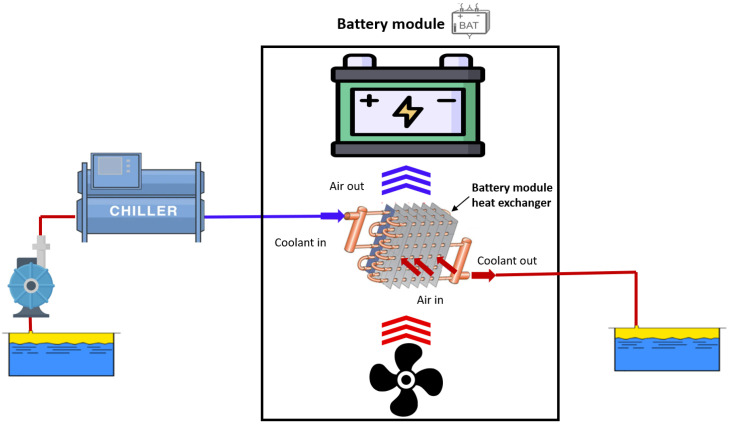
Framework of the battery cooling system.

Figure 2 describes the functioning of the battery module, which is composed of the battery cells, a heat exchanger, and a fan placed underneath. First, the coolant passes through a chiller, in which it reaches 25 °C, and then, thanks to an air to liquid heat exchanger, it cools the air that is conveyed to the battery cells with a fan.

In the first stage of this research the cooling system section was modeled without the batteries because all sixteen battery modules were not available at first; therefore, the cooling system without batteries was the only prototype available for experimental validation. The proposed hydraulic model was validated via comparing simulations and experimental results that were carried out via ultrasonic clamp-on flow measurements. In the second stage, the single battery module was hydraulically characterised, and the previous validated model was completed with battery modules. Thanks to the completed model, it was possible to evaluate the unbalance of flow rates in the individual battery modules and then choose the best way to achieve the balance.

In this work, two solutions were proposed: The first involves the use of calibrated orifices in the branches of the cooling system with greater flow rate. The second involves a layout modification without introducing secondary losses. Finally, a comparison between the two solutions was carried out.

### 2.2. Ultrasonic Clamp-On Flow Measurements

Experimental flow measurements were carried out with the ultrasonic clamp-on sensor Keyence FD-Q32C for DN32 pipes, powered by a 24 V power supply. This sensor measures the time it takes to transmit a signal ultrasound from the emitter to the receiver. If the flow rate increases, the signal is accelerated, that is, less time is required for transmission from the emitter to the receiver. By employing the correlation between duration and flow velocity, the sensor measures the instantaneous flow rate [32].

**Figure 3:**
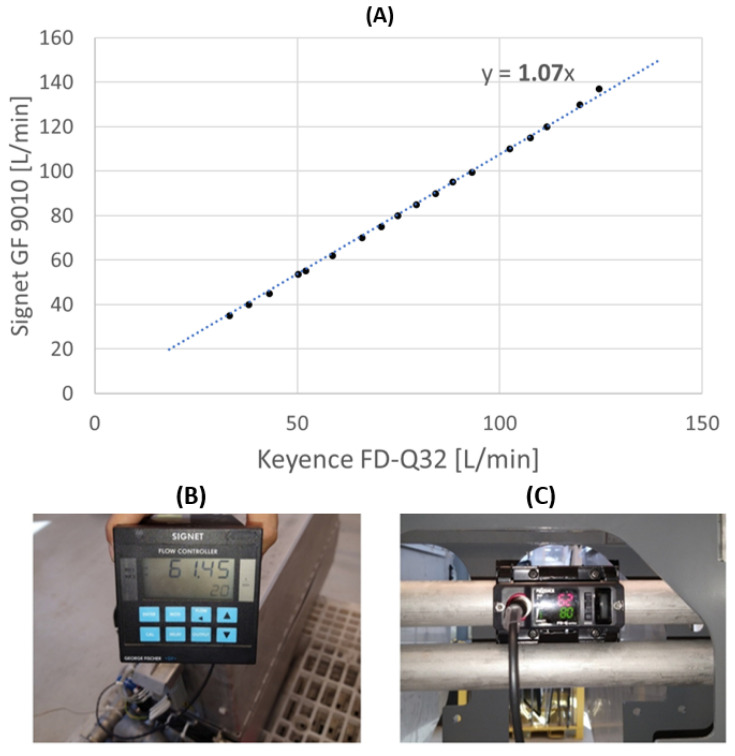
(**A**) Least-squares linear regression of the data obtained with the two sensors; (**B**) George Fischer measurement; (**C**) Keyence measurement with adjusting flow rate span.

A clamp-on type sensor was chosen because it allows us to easily evaluate the flow rate of the various branches of the system without modifying the system structure, since it was installed outside the pipe for a completely non-wetted measurement. This prevented any risk of adverse effects on the liquid and eliminated the need for piping work. On the other hand, this sensor must be calibrated by adjusting the flow rate signal with a calibration coefficient, whose value was obtained from the comparison with the measurement carried out by a turbine flowmeter (Signet Flow Controller GF George Fischer 3-9010.111) that was positioned downstream of the pump and has an accuracy of ±0.5% [33]. The optimal value of the adjusting flow rate, equal to 1.07, was calculated as the slope of the last-squares linear regression of the data obtained with the two sensors, as shown in Figure 3. In this case, from the tests carried out, the optimal value of the adjusting flow rate span was found to be 1.07.

### 2.3. Numerical Model of the Prototype Available

**Figure 4:**
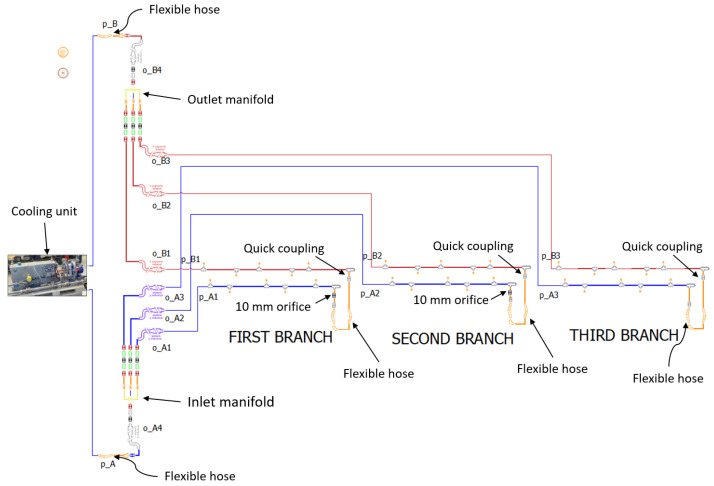
Simcenter AMESim model of the prototype available for testing.

The first analyses were carried out on the available prototype that included the hydraulic system without the battery modules. This is because once the batteries were mounted in the battery box enclosure it was not possible to measure the flow rate in the various branches, since there is not enough space left to mount the clamp-on flow sensor. The hydraulic system object of this research is a closed-cycle circuit with three branches in parallel of different lengths which mainly consists of a cooling unit (pump and tank), inlet and outlet manifolds, delivery and return pipes, flexible hoses, quick couplings, and an orifice with a diameter of 10 mm and a thickness of 6 mm in the first and second branches resulting from a preliminary sizing of the system. The circuit was implemented in Simcenter AMESim, a multi-domain, lumped parameter simulation software, suitable for performing simulations of the system as a whole, in which the arrangement of components refers to that in the physical prototype (Figure 4).

**Figure 5:**
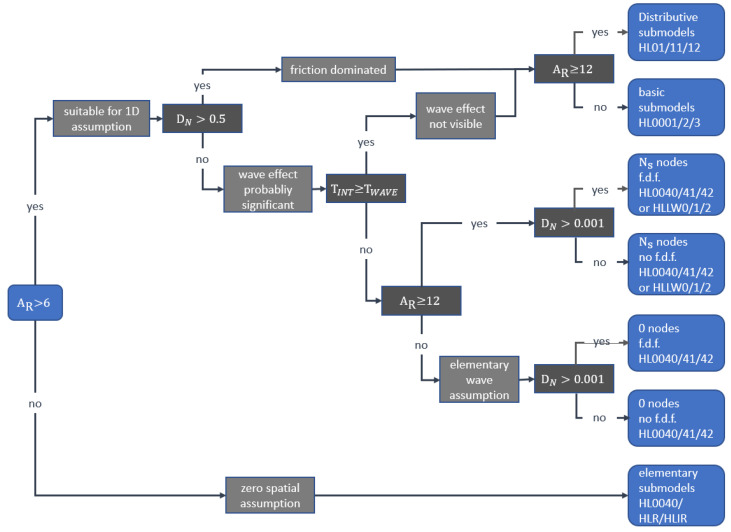
Flow chart for the choice of submodels of the hydraulic lines [34].

The mainly used libraries were Thermal Hydraulic and Thermal Hydraulic Resistance. The branches of different lengths ($l_{1}<l_{2}<l_{3}$) were modeled using the thermal–hydraulic modular piping that includes straight pipes, direction changes, and diameter changes. This submodel calculated the pressure drops while taking into account the compressibility of the fluid and expansion of the pipe wall with pressure. The heat exchange and the influence of temperature on the fluid viscosity were also considered. The methodology described by the flowchart in Figure 5 was followed in order to select the AMESim submodels of the pipelines of the cooling system. This line selection method considers the parameters of the line and the fluid properties, and then it provides an analysis involving the following values:

- The aspect ratio $A_{r}$ is the ratio between the length of the section *l* and the hydraulic diameter $d_{h}$:
  (1) $$A_{r}=l/d_{h}$$
- The dissipation number $D_{n}$ is defined as:
  (2) $$D_{n}=\frac{4l\nu }{cd_{h}^{2}}$$
  where ν is the kinematic viscosity and *c* is the speed of sound;
- The wave travel time $T_{wave}$ is the time that pressure disturbance takes to cross the pipe:
  (3) $$T_{wave}=l/c.$$

The coolant used was the Antifrogen N-39, produced by Clariant, which consists of a mixture of 60% water and 40% ethylene glycol added with corrosion inhibitors. It was modeled by using the Media Property Assistant tool, in which parameters such as density, viscosity, and heat capacity, obtained from the datasheet (available on the supplier’s website [35]) were inserted. The cooling unit was modeled as a super component which mainly contains a 60 L tank and a centrifugal pump with an impeller with a diameter of 139 mm.

**Figure 6:**
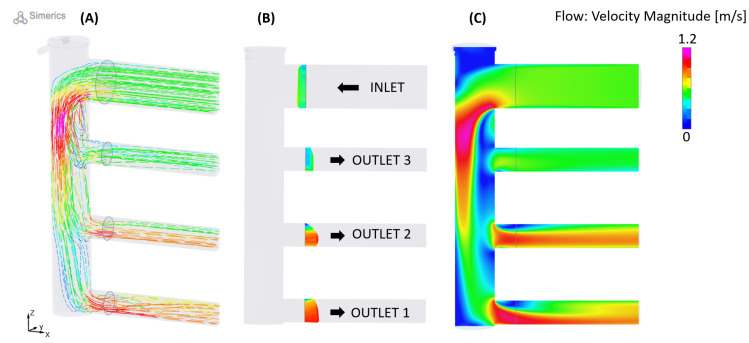
The 3D CFD simulation of flow rate distribution in the manifold. (**A**) Streamlines view; (**B**) vectors view; (**C**) contour view.

The flow was then split by a three-way manifold. This component was characterized using another tool called Simerics MP+, a commercial 3D CFD simulation software, in order to evaluate how the inlet flow rate (138 L/min) was distributed to the manifold outlets. In this study, a steady-state flow analysis was performed that includes a standard k−ϵ turbulence model with a Converge Criterion of $10^{-4}$. The boundary conditions were an inlet volumetric flux of 138 L/min and an outlet pressure of 101,325 Pa. Figure 6 provides a synthesis of the results derived from this 3D CFD simulation.

**Figure 7:**
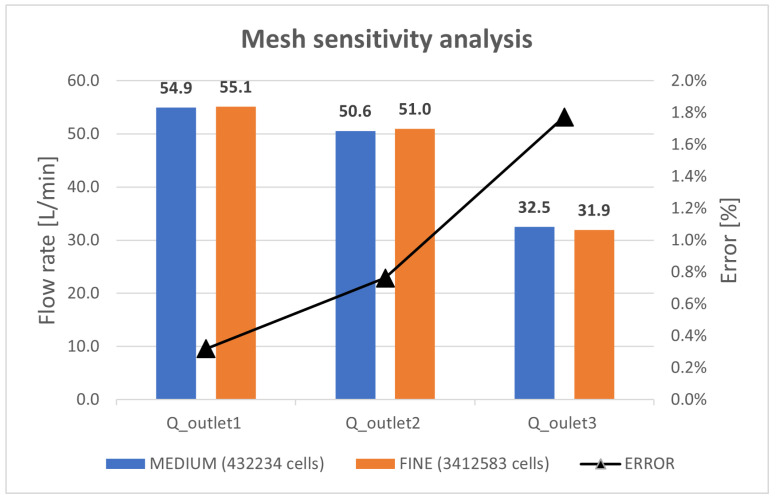
Mesh sensitivity analysis.

Mesh sensitivity analysis was performed for the outlet flow rates $Q_{outlet1}$, $Q_{outlet2}$, $Q_{outlet3}$ where medium mesh and fine mesh had, respectively, $0.43\times 10^{6}$ and $3.4\times 10^{6}$ cells. The results of the mesh sensitivity analysis are shown in Figure 7, from which it is clear that a good level of approximation can be obtained with a medium mesh with a lower computational effort. A workstation, equipped with a 64 GB RAM and Intel^®^ Xeon^®^ CPU E5-2699 v3 2.30 GHz processor, was used to perform the analyses. The calculation times in the case of medium mesh and fine mesh using 8 cores are, respectively, 468 s and 1093 s.

**Figure 8:**
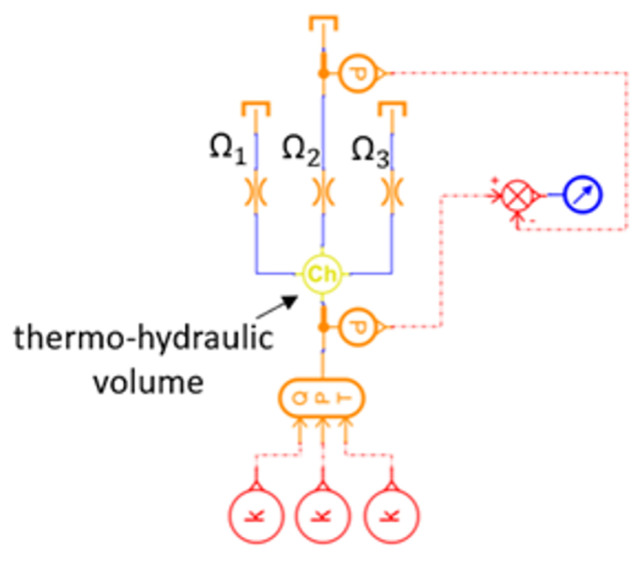
Manifold lumped parameter model.

The manifold was then modeled in Simcenter AMESim (Figure 8) by means of a thermo-hydraulic volume with one inlet and three outlets, and by inserting an equivalent orifice at each outlet. The cross-sectional area of these orifices (respectively, $\Omega_{1}$, $\Omega_{2}$, and $\Omega_{3}$) was evaluated in order to obtain the same flow distribution found in the 3D CFD characterization, as described below.

The three orifices are in parallel [36]; therefore, Equation (4) holds:

(4) $$\Omega_{eq}=\Omega_{1}+\Omega_{2}+\Omega_{3}.$$

From the known orifice Equation [36], knowing $Q_{inlet}$ and Δp, it is possible to obtain $\Omega_{eq}$:

(5) $$\Omega_{eq}\left[{\mathrm{mm}}^{2}\right]=\frac{Q_{inlet}\left[L/\min\right]}{18.97\cdot C_{f}}\cdot \sqrt{\frac{\rho }{2\Delta p[bar]}}$$

where ρ is the coolant density at 25 °C, equal to 1050 kg/m${}^{3}$, and $C_{f}$, equal to 0.611, is the von Mises’ theoretical value for a circular sharp edge orifice [36]. Finally, from the flow rate ratios $Q_{outlet1}/Q_{outlet2}$ and $Q_{outlet1}/Q_{outlet3}$ and from Equation (4) it is possible to derive the sections $\Omega_{1}$, $\Omega_{2}$, and $\Omega_{3}$, respectively, equal to 781 mm${}^{2}$, 1221 mm${}^{2}$, and 1343 mm${}^{2}$.

## 3. Results for the Model Validation

In this section, the numeric hydraulic results are shown.

**Figure 9:**
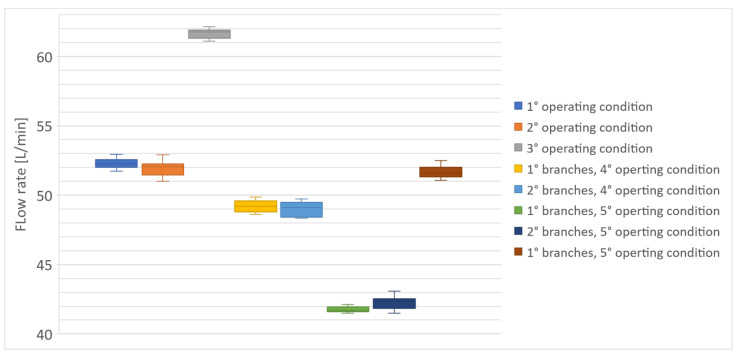
Box plots of the experimental measurements. Number of tests repeated for each case: 10.

Although the system works in a single operating point, to better test the model, it was validated by comparing the numerical results with the experimental ones in three different operating conditions, as shown in Table 1. The first three operating conditions provided the passage of fluid only in one branch at a time and excluded the other two. In the fourth operating condition the third branch was excluded, whereas in the fifth condition, which was the only condition that simulated the real operating conditions of the system, the coolant flowed in all branches at the same time. The experimental measurements were repeated ten times and are reported in the box-plots shown in Figure 9. From the last condition, it is possible to notice, as expected, that when the first two branches are shorter, they had a lower volumetric flow rate than the third due to the presence of the 10 mm orifices.

Table 1 also summarizes the comparison between the numerical results and the mean values of the experimental results obtained by the Keyence FD-Q32C sensor in all operating conditions.

The results show that in most cases the error falls within the uncertainty range of the flow sensor, equal to 1 L/min [37]. For the third branch only, a slightly higher error was encountered. Nevertheless, the model is considered sufficiently reliable and can be used for further investigations and optimization of the system.

## 4. Complete Hydraulic Model with Battery Modules

### 4.1. Battery Cooling System Description and Characterization

**Figure 10:**
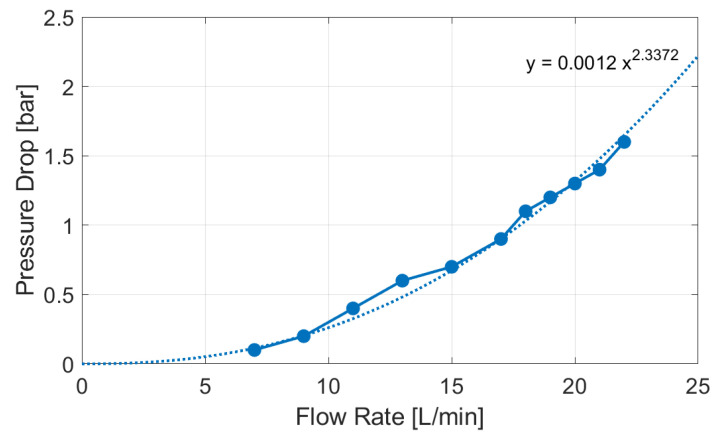
(Q÷Δp) curve of the battery module heat exchanger.

**Figure 11:**
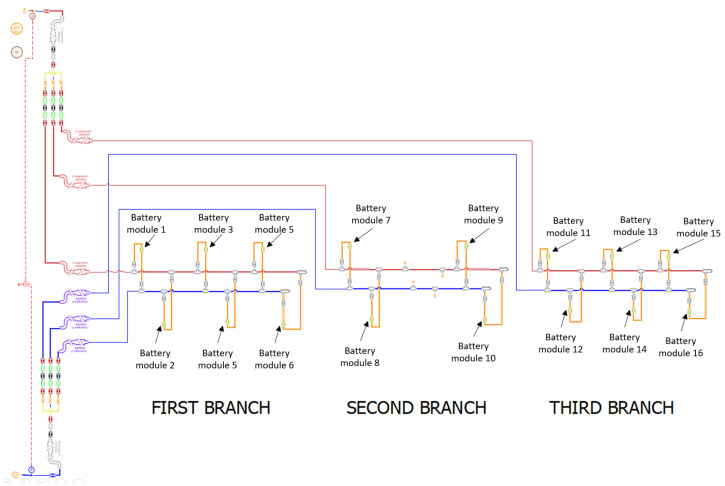
Numerical model with battery modules.

The previously validated model was extended by implementing a submodel that introduced the pressure drop due to the air to liquid heat exchanger, integrated in the battery module. The heat exchanger was experimentally characterized as a pressure loss on the test bench. Figure 10 shows the (Q−Δp) curve that was derived from these experimental data. This curve was assigned to an orifice that simulates the hydraulic behavior of the battery module and then was implemented using the previous validated numerical model (Figure 11).

### 4.2. Hydronic Balancing

**Figure 12:**
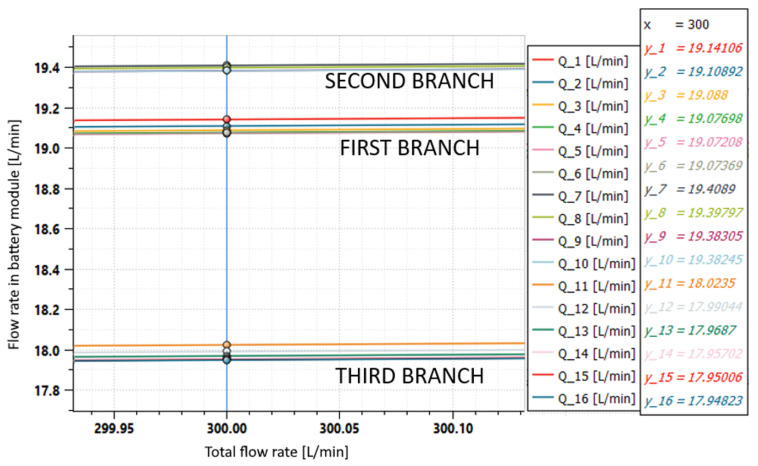
Flow rates in the individual battery modules for an inlet flow of 300 L/min.

As previously said, each of the 16 battery modules that compose the electrical energy storage system must be refrigerated with a specific flow rate at a specific temperature. The system architecture (Figure 1) is made by three branches with the modules arranged in parallel, in order to favor an equal distribution of the inlet flow Q of the coolant and, therefore, to ensure the same thermal conditions for all users. Even if all the users are in parallel, from a physical point of view these are arranged in sequence in order to reach each battery by a line section of different length. The first and third branches serve six users while the second serves only four users. As can be seen from Figure 12, these conditions favor the unbalancing of the flow rates; therefore, actions are needed to obtain the optimum condition of hydronic balancing:

(6) $$Q_{1}≅Q_{2}≅\ldots ≅Q_{16}.$$

In this case two solutions are proposed, the first provides calibrated orifices in the branches with greater flow rate; the second involves a layout modification.

#### 4.2.1. Solution 1: Calibrated Orifices

**Figure 13:**
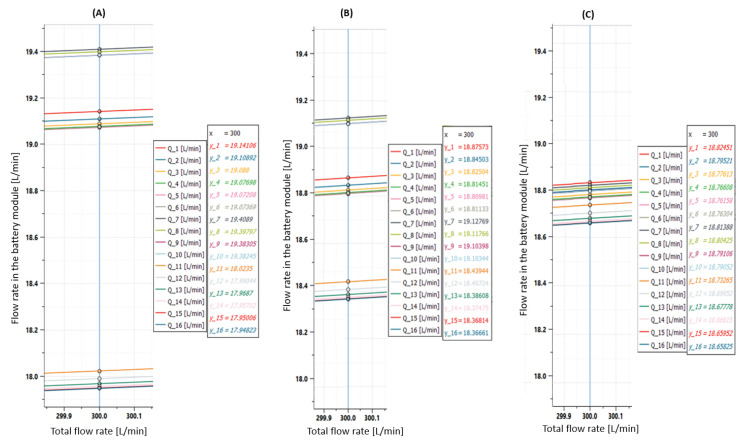
(**A**) Flow rates without calibrated orifices. (**B**) Flow rates with 10 mm orifice in the first and second branches. (**C**) Flow rates with 9 mm orifice in the first branch and 8.5 mm orifice in the second branch.

From the diagram of the individual flow rates (Figure 12 and Figure 13A), it can be seen that the flow rate in the first two branches was higher than the one in third branch. In order to balance the flow rates, the calibrated orifices can be placed upstream of the battery modules of the first two branches. Figure 13B shows the results obtained when using 10 mm orifices in the first two branches of the cooling system, while Figure 13C shows the results obtained when using 9 mm orifices in the first branch and 8.5 mm orifice in the second branch. With these measures, a fairly good hydronic balance was achieved; the difference between the higher and the lower flow rate was reduced by 90%.

#### 4.2.2. Solution 2: Layout Modification

Assuming the pressure drop across a single battery module can be described as the pressure drop occurring in an equivalent orifice with a section Ω, the flow rates that circulate in the three branches of the hydraulic system were evaluated according to Equation (7):

(7) $$Q=\sqrt{\frac{\Delta p}{R}}$$

where *R* indicates the hydraulic resistance introduced by the equivalent orifice:

(8) $$R=\frac{\rho }{2C_{f}^{2}\Omega^{2}}.$$

Since the three branches were in parallel, the pressure drop across the hydraulic circuit was always equal to $\Delta p_{A,B}$, and, since all the battery modules are the same, the sections of the equivalent orifices with which they were replaced were equal. According to the definition of the orifices in parallel, the first and the third branches, which deliver coolant to six battery modules, had an equivalent orifice section of 6Ω, while the second branch, with four battery modules, had an equivalent orifice section of 4Ω. From Equations (7) and (8), it is possible to evaluate the flow rates in the three branches $Q_{I}$, $Q_{II}$, and $Q_{III}$ as follows:

(9) $$Q_{I}=Q_{III}=C_{f}\cdot 6\Omega \sqrt{\frac{2\Delta p_{A,B}}{\rho }}$$

(10) $$Q_{II}=C_{f}\cdot 4\Omega \sqrt{\frac{2\Delta p_{A,B}}{\rho }}.$$

Therefore:

(11) $$\frac{Q_{I}}{Q_{II}}=\frac{Q_{III}}{Q_{II}}=1.5$$

which is consistent with the results found before.

Regarding the pressure drop of the three branches it can be observed that (Figure 1):

(12) $$\Delta p_{A,B}=\Delta p_{A1,B1}+\Delta p_{A,A1}+\Delta p_{B1,B}$$

(13) $$\Delta p_{A,B}=\Delta p_{A2,B2}+\Delta p_{A,A2}+\Delta p_{B2,B}$$

(14) $$\Delta p_{A,B}=\Delta p_{A3,B3}+\Delta p_{A,A3}+\Delta p_{B3,B}.$$

Assuming:

(15) $$\Delta p_{A,A1}+\Delta p_{B1,B}=\Delta p_{1}$$

(16) $$\Delta p_{A,A2}+\Delta p_{B2,B}=\Delta p_{2}$$

(17) $$\Delta p_{A,A3}+\Delta p_{B3,B}=\Delta p_{3}$$

it is possible to rewrite Equations (12)–(14) as:

(18) $$\Delta p_{A,B}=\Delta p_{A1,B1}+\Delta p_{1}$$

(19) $$\Delta p_{A,B}=\Delta p_{A2,B2}+\Delta p_{2}$$

(20) $$\Delta p_{A,B}=\Delta p_{A3,B3}+\Delta p_{3}$$

where, from the Darcy–Weisbach equation:

(21) $$\Delta p_{i}=\frac{\lambda_{i}l_{i}}{d_{h}}\frac{\rho Q_{i}^{2}}{2A^{2}}\;\;with\;\;i=1,2,3\;\;\left(for\;Q,\;i=I,II,III\right)$$

in which

- λ is the friction factor for a relative unitary length stretch of pipeline $l/d_{h}$; it depends on the Reynolds number and the relative roughness of the inner surface of the pipe;
- $d_{h}$ is the hydraulic diameter;
- *l* is the length of the pipeline section for each branch;
- *A* is the section of the pipe.

Analyzing Equation (21), from Equation (11) results:

(22) $$Q_{I}≅Q_{III}>Q_{II}.$$

In addition, given the arrangement of the branches:

(23) $$l_{1}<l_{2}<l_{3}$$

while, for high Reynolds numbers (as in the present case, where Re is of the order of $10^{6}$), λ is almost constant.

Given the relations (22) and (23), and since $\Delta p_{i}$ depends on the square of the flow rate, while only linearly on the length of the stroke, it is observed that:

(24) $$\Delta p_{3}>\Delta p_{1}>\Delta p_{2}$$

from which, given (18)–(20), it follows that:

(25) $$\Delta p_{A2,B2}>\Delta p_{A1,B1}>\Delta p_{A3,B3}$$

and finally, given (7), results:

(26) $$Q_{7,8,9,10}>Q_{1,2,3,4,5,6}>Q_{11,12,13,14,15,16}$$

which is consistent with the results shown in Figure 12.

From the previous considerations it appears that if the $\Delta p_{i}$ are the same, the flow rates in the individual modules are also equal; therefore, in order to improve the balancing of the flow rates without introducing further pressure drops, Equation (21) must be considered, in which all terms are constant, except for *l* and *Q*.

**Figure 14:**
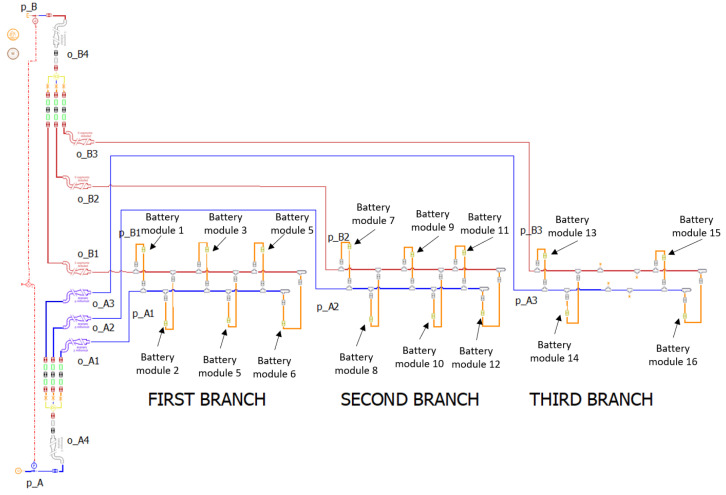
Layout modification: the branch with fewer users is placed as last.

**Figure 15:**
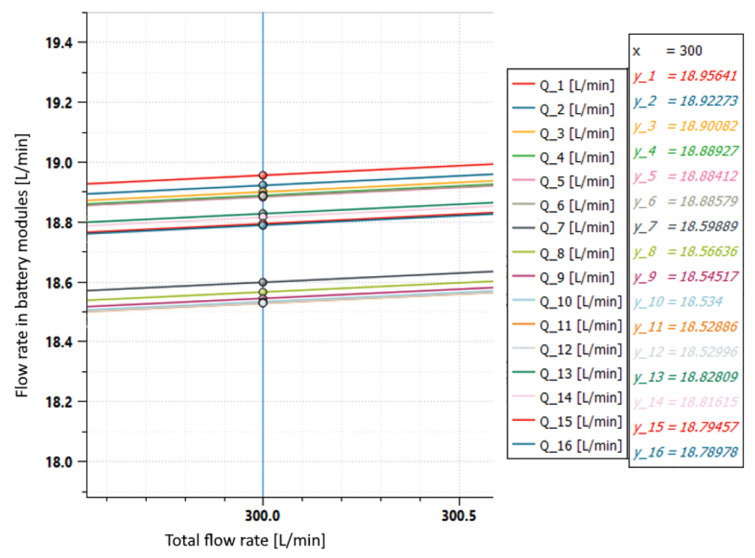
Flow rates in the individual battery modules in case of layout modification.

*Q* depends on the number of modules placed in a branch, as can be seen from (9) and (10), while *l* depends on the position in which the modules are arranged. To equal the $\Delta p_{i}$ it is necessary to increase *l* when *Q* is small; in other words, the branch with fewer users must be placed as far away as possible as well as in the model in Figure 14. In this condition it is possible to see the results in terms of flow rate in the individual modules in Figure 15.

Thanks to this solution, a maximum flow rate imbalance of 0.4 L/min was obtained, which was much lower than the maximum flow rate imbalance of 1.5 L/min of the initial layout. In this case the difference between the higher and the lower flow rate was reduced by 73%.

#### 4.2.3. Comparison between the Two Solutions

In terms of flow rates, the solution with the calibrated orifices involves a better balance than the layout modification. Indeed, while the first reduces the imbalance by 90%, the second is limited to 73%. In contrast, in terms of overall pressure drop between the inlet manifold and the outlet manifold $\Delta p_{A,B}$ (Figure 1), the first solution provided a $\Delta p_{A,B1}$ of 1.47 bar, while the second, since it introduced secondary losses due to orifices, provided a higher $\Delta p_{A,B2}$ of 1.64 bar. This difference in pressure drops, multiplied by the nominal flow rate Q of 300 L/min, caused a global power saving $P_{s}$ for the whole system of 585 W.

## 5. Conclusions

In this paper, a lumped parameter model of the three branches liquid cooling system of a battery pack for railway traction was initially validated. The validation was carried out in two phases. First, a model of a cooling system without batteries was used, in which the numerical results were compared with the experimental results obtained using an ultrasonic clam-on type flow sensor. The results obtained showed a maximum error of 2% in the first two branches, while, in the third branch, there was a slightly higher error which will be investigated by inserting additional pressure transducers in the next battery box prototype. Then, the battery modules, previously characterized on a test bench, were also included in the model. The validated model was used to find the hydronic balancing condition among all the battery modules. To distribute the flow equally, two solutions were proposed. The first involved calibrated orifices upstream of the modules with greater volumetric flow rates, while the second modified the layout by placing the branch with fewer users in the last position. Finally, the two solutions were compared to evaluate the energy savings obtained by using the second solution instead of the first. From the comparison, using the second solution, a power saving for the pump of almost 600W was estimated.

This power saving method is based on the equalization of the flow rates without introducing secondary losses. This is possible if the branch in which there is the lowest flow rate, the one with the least utilities, is also the longest one. Thus, the product of the two terms l and Q in the Darcy–Weisbach equation tends to be the same for every branch.

This model represents a significant advantage and provides great support in the optimization phase of the cooling system in terms of energy saving and rapid and low-cost experimentation, thus increasing the efficiency of the R&D phase in product manufacturing. Indeed, thanks to this approach it has been possible to compare several solutions without building expensive prototypes, both when considering the calibration of the orifices and the assembly of the system.

In this case, the main challenge facing liquid BTMS was to obtain a criterion for energy savings derived only from the hydraulic system. In order to achieve this, different flow measurements were necessary at different points in the system. The use of a clamp-on type sensor made the experimental campaign easier and more flexible, since with just one sensor it was possible to detect the flow rate in different branches without making any changes to the system.

Future developments will provide the implementation of a model for the thermal dissipation of the batteries in order to create a complete digital twin of the cooling system of the battery thermal management that will allow us to perform numerical tests aimed at finding the best cooling method to reduce energy consumption for safe, sustainable, and comfortable collective mobility.

## Figures and Tables

**Table 1:** Comparison between the numerical and the experimental results.

Operating Condition	First Branch			Second Branch			Third Branch		
	Numeric (L/min)	Exp. (L/min)	Error %	Numeric (L/min)	Exp. (L/min)	Error %	Numeric (L/min)	Exp. (L/min)	Error %
**1**	53	52	2	0	0	0	0	0	0
**2**	0	0	0	53	52	2	0	0	0
**3**	0	0	0	0	0	0	67	62	8
**4**	50	49	2	50	49	2	0	0	0
**5**	42	43	2	42	42	0	54	52	4

## Data Availability

Data available on request due to restrictions, e.g., privacy or ethical.

## Nomenclature

*A*	Pipe cross-sectional area ($m^{2}$)	*Q*	Volumetric flow rate ($m^{3}/s$)
$A_{r}$	Aspect ratio (-)	*R*	Hydraulic resistance ($Pa\;s^{2}/m^{6}$)
*c*	Speed of sound (m/s)	Re	Reynolds number (-)
$C_{f}$	Orifice coefficient (-)	rr	Relative roughness (-)
$d_{h}$	Hydraulic diameter (m)	$T_{wave}$	Wave travel time (s)
$D_{n}$	Dissipation number (-)	λ	Friction factor (-)
*l*	Length of a pipeline (m)	ν	Kinematic viscosity ($m^{2}/s$)
*p*	Pressure (Pa)	ρ	Density ($kg/m^{3}$)
*P*	Power (-)	Ω	Orifice cross-sectional area ($m^{2}$)
